# Use, Usability, and Experience Testing of a Digital Health Intervention to Support Chronic Kidney Disease Self-Management: Mixed Methods Study

**DOI:** 10.2196/75845

**Published:** 2025-06-19

**Authors:** Courtney J Lightfoot, Thomas J Wilkinson, Roseanne E Billany, Gurneet K Sohansoha, Noemi Vadaszy, Ella C Ford, Melanie J Davies, Thomas Yates, Alice C Smith, Matthew P M Graham-Brown

**Affiliations:** 1 Department of Population Health Sciences University of Leicester Leicester United Kingdom; 2 NIHR Leicester Biomedical Research Centre Leicester United Kingdom; 3 Diabetes Research Centre University of Leicester Leicester United Kingdom; 4 Department of Cardiovascular Sciences University of Leicester Leicester United Kingdom; 5 School of Humanities and Social Sciences Leeds Beckett University Leeds United Kingdom; 6 Department of Renal Medicine University Hospitals of Leicester NHS Trust Leicester United Kingdom

**Keywords:** chronic kidney disease, digital health, engagement, patient activation, self-management, user experience

## Abstract

**Background:**

We developed My Kidneys & Me (MK&M), a digital health intervention (DHI) that delivers specialist health and lifestyle education, to improve self-management in people with chronic kidney disease (CKD).

**Objective:**

We aimed to explore the uptake and usability of MK&M alongside patient experiences of using MK&M.

**Methods:**

Adult patients with CKD stages 3-4 were recruited from 26 hospital kidney services in England. Overall, 420 participants were randomized 2:1 to the intervention (MK&M) or control (usual care) group. Uptake and usage data were collected from the MK&M program. Perceived usefulness of the MK&M sessions and features were collected via web-based surveys (scores were rated out of 10, where 0=“not useful at all” and 10=“very useful”). Qualitative (semistructured and think-aloud) interviews were used to explore participants’ experiences of using and engaging with MK&M. Usage metrics were assessed using descriptive and frequency analyses. Qualitative data were analyzed using thematic analysis.

**Results:**

Overall, 280 participants were randomized to receive the MK&M intervention (age: mean 60.8, SD 12.8 y; n=161, 57.5% male; eGFR: mean 38.9, SD 18.5 mL/min/1.73 m^2^). Of those, 225 (80.3%) participants activated and used their MK&M account. The median number of log-ins per person was 10.0 (IQR 4.0-28.0). The median time per log-in was 12 (IQR 7-25) minutes. “The kidneys” was the most accessed session (152/225, 67.6%). The educational sessions were the most valued and engaging content, while health and symptom trackers were the least used features. All sessions received scores ≥7 out of 10 for perceived usefulness, with “Kidney disease and general health” considered most useful (score=8.7/10). Goal setting for health behaviors was considered the most useful tracker (score=8.5/10) and symptoms the least (score=6.7/10). Overall, 33 participants were interviewed (n=6, 18% think-aloud; n=27, 81% semistructured). Themes relating to use, usability, and engagement with MK&M were identified. MK&M was well-received, with participants reporting that the user interface was easy to use, with clear and logical navigation and appropriate presentation of information. Learning sessions were more widely accessed and used than the action (“How to...”) sessions, with participants highlighting not having enough time to engage with all the MK&M content during the study period. MK&M users had positive experiences of using the program; however, there was ambivalence regarding content and features, which could be explained by personal preference rather than usability issues. Participants had a desire for continued learning and perceived the relevance of MK&M to be greater with time and disease progression.

**Conclusions:**

The MK&M DHI was well-received and used by the participants. Our findings show that a wide range of people with CKD, including older adults, are capable and willing to use DHIs for kidney health. Identification of real-life use and usability issues will help refine MK&M to improve the content and delivery for clinical implementation.

## Introduction

### Background

Chronic kidney disease (CKD) affects approximately 10% of the global population [1] and is associated with significant morbidity, disease burden, and excess mortality [2]. Most individuals living with CKD have early-stage disease and are usually managed in primary care by their general practitioner or family physician [3]. As a result, people with early-stage CKD do not have access to the same specialist support, education, and training as those looked after by specialist kidney teams and are not well informed about their condition and its implications [4,5]. Improving people’s knowledge of CKD and its complications is paramount for optimal management to prevent disease progression and associated health problems [6].

Over the last decade, self-management interventions for CKD have gained popularity [7], in part due to the increasing recognition of the importance of incorporating patients and their families in managing long-term health conditions to improve outcomes [8]. One approach to delivering self-management education and support is the use of digital technology and tools. Digital health interventions (DHIs) have the potential to address self-management educational and support needs as they are largely accessible by most of the population, can be highly effective, and are delivered at low cost [9]. Despite DHIs becoming more readily available for people with CKD, few are theory based, and the development strategies and processes are unclear [10]. Adoption of appropriate methods is essential when developing DHIs to ensure effective implementation [11]. In addition, the codevelopment of DHIs with patient partners is crucial for their success, particularly in combination with usability testing [12,13], to ensure that relevant needs, priorities, and issues are addressed.

To support self-management in people with nondialysis CKD, we codeveloped “My Kidneys & Me” (MK&M), an evidence- and theory-based DHI that provides tailored, interactive information and support to improve CKD awareness and understanding, health knowledge, health-promoting behaviors, and confidence [14,15]. MK&M was codeveloped with kidney health care professionals and researchers and specialists in the development of complex interventions and digital health, in partnership with individuals living with CKD and their relatives. Intervention Mapping was used as a framework to guide the development processes of MK&M, in combination with several theory-informed intervention methods and practical strategies to facilitate behavior change. The MyDESMOND platform was used to host the MK&M DHI; MyDESMOND is an award-winning DHI on a quality-assured platform, which has been successfully developed and implemented [16] and is known to be well-accepted and effective [17]. The MK&M DHI has been evaluated in a multicenter randomized controlled trial (RCT), Self-Management Intervention through Lifestyle Education for Kidney Health (SMILE-K), which demonstrated positive effects, particularly for those with low levels of activation (knowledge, skills, and confidence in managing their own health) and in those who used the program more [10].

Similar theory-informed DHIs for CKD self-management have been co-designed, using frameworks to guide development, in the United Kingdom [18,19], Canada [20], and the United States [21]. Similarities and differences between MK&M and these DHIs have been described elsewhere [14]. Kidney Beam, a DHI addressing physical and emotional well-being [18,19], has demonstrated improvements in mental health–related quality of life in a multicenter RCT [22]. In a preliminary evaluation, My Kidneys My Health [20] has been shown to provide accessible content and tools that may improve self-efficacy and support in CKD self-management [23], but a larger trial is warranted. Qualitative exploration of a mobile app to support CKD self-management [21] suggested that individuals with CKD and their health care providers believe that the app can enhance CKD self-management by facilitating patient–health care provider communication and enabling self-care activities; however, its effectiveness on patient outcomes has not yet been evaluated.

### This Study

Alongside demonstrating the efficacy of DHIs, the use of and engagement with DHIs are considered important when evaluating their effectiveness [24,25] and may be regarded as a prerequisite for the intervention to achieve positive outcomes [26]. Engagement, defined as users’ regular interaction with a part or all of the DHI [27], has been typically conceptualized as “use” [24], with a focus on temporal patterns (eg, frequency and duration) and depth (eg, use of specific intervention content) [28,29]. Although the primary aim of the SMILE-K trial was to determine the efficacy of MK&M on patient activation and self-management knowledge and behaviors [10,15], a secondary aim was to gain insight into participants’ use and experiences of MK&M. In this paper, we aimed to report the use metrics and patient experiences of using MK&M during a 20-week evaluation as part of the SMILE-K RCT.

## Methods

### Overview

This analysis comprises data collected as part of the SMILE-K trial (ISRCTN18314195). The SMILE-K study protocol [15], pilot study [30], and primary findings [10] are published elsewhere. As this paper reports on the use metrics and user experience of using MK&M, the data presented are from the intervention group only.

### Study Design and Setting

The SMILE-K trial was a single-blinded 20-week prospective, mixed methods, multicenter randomized controlled, parallel-group trial recruiting patients from 26 hospital sites across England, between May 2021 and December 2022. All study processes were conducted remotely due to the COVID-19 pandemic and for pragmatic evaluation. Full details of the study processes are published elsewhere [15].

### Ethical Considerations

This study was fully approved by the Research Ethics Committee-Leicester South on November 19, 2020 (17/EM/0357). All participants gave web-based informed consent for the trial; interview participants provided additional consent before the commencement of the interviews. The study was conducted per the Declaration of Helsinki. The trial was sponsored by the University of Leicester.

The study was prospectively registered on ISRCTN Registry (ISRCTN18314195) on December 18, 2020. Data were anonymized or deidentified.

### Participants

#### Overview

Eligible participants were aged ≥18 years with CKD stages 3 to 4 (estimated glomerular filtration rate [eGFR] 15-59 mL/min/1.73 m^2^) or diagnosed with a progressive kidney condition with an eGFR >60 mL/min/1.73 m^2^ (eg, polycystic kidney disease) not receiving renal replacement therapy. Eligible participants were invited via a letter and promotional flyer. Interested participants were asked to contact the lead research team via email to receive a web-based participant information sheet and consent form. Following consent, participants were sent a link to a web-based survey collecting baseline outcome measures. Participants were subsequently randomized 2:1 into intervention or control groups, stratified by age (≤63 years and >63 years), using computer-generated randomization [15]. Researchers at the lead site were not concealed from the allocation sequence, and participants were not blinded to group allocation. The participants in the intervention group received the MK&M program, as detailed in a subsequent section, and the participants in the control group received usual care and were asked to maintain their habitual lifestyle activities and follow their clinical care plans. The participants in the control group were not provided with the MK&M program during the trial but were provided with the intervention on study completion.

All trial participants were eligible for the qualitative substudy. Interview participants were purposively selected to capture a diverse range of participant characteristics to ensure a representative population. Patient and public (PPI) members, who live with CKD (stages 3-4) and meet the trial eligibility criteria but were not involved in the trial, were invited to participate in MK&M think-aloud interviews.

#### The MK&M Intervention

Participants randomized to the intervention group received immediate access to the MK&M program with a unique user log-in. Hosted on the MyDESMOND platform [16], the MK&M program is a web-based application that was systematically developed as a theory- and evidence-based digital self-management intervention for people with nondialysis CKD; it is designed to provide holistic CKD-specific self-management education, support, and guidance for people living with mild to moderate CKD. The development of MK&M and its features are discussed in detail elsewhere [14,15].

**Figure 1:**
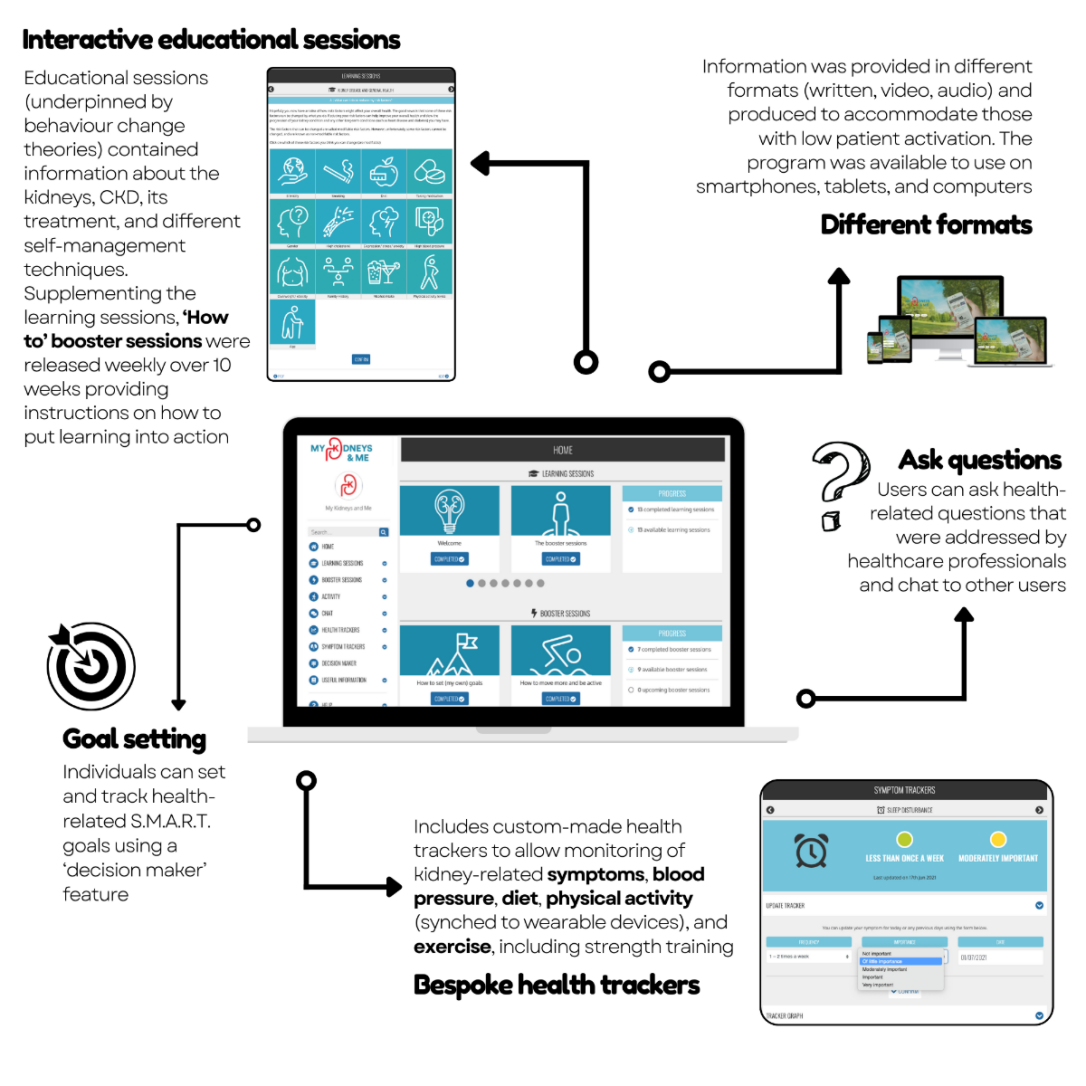
My Kidneys & Me dashboard and features (reproduced from Lightfoot et al [10]).

The MK&M program provides a structured 10-week program with tailored, interactive information and support to improve (1) patient activation: awareness and understanding of CKD, health-related knowledge, skills to engage in health-promoting behaviors, and confidence in managing their health and (2) related CKD self-management knowledge and behaviors, including symptom management, lifestyle behaviors, and physical function [10,14,15] (Figure 1). This was achieved through the provision of content and materials designed to increase CKD knowledge and patient activation, reduce health risks, manage symptoms, and improve physical function. The program components developed comprised educational and behavior change action (“How to...”) sessions; health trackers (eg, monitoring blood pressure, symptoms, and exercise); ability to link up activity tracking devices (eg, Fitbit, Garmin, and Google Fit); goal-setting features; and forums for social support. Educational “Learn about” sessions provided information about kidneys, CKD, its treatment, symptoms, associated health risks, and lifestyle-related factors (eg, diet and physical activity). Behavior change–focused “How to...” sessions were designed to provide practical strategies to assist individuals with making small modifications to improve their health and lifestyle behaviors, and support individuals to put their learning (from the “Learn about” sessions) into action. The “How to...” sessions also contained information about how to set goals, self-monitor health and behaviors, and create action plans, as goal setting is a motivator to facilitate health and lifestyle behavior changes. Information provided in the “Learn about” and “How to...” sessions followed the same structure but was presented in a variety of formats and media (ie, text, graphics, animation, and video) and used quizzes as a way to test knowledge. As new content was released weekly for 10 weeks, 20 weeks was selected as the primary end point to allow participants time to review the educational content, put learning into action, and explore other features on the program. As part of the MK&M program, participants received automated reminder emails when new content (ie, the “How to...” sessions) was available to them. Participants also received automated reminder emails if they had not logged in after 7, 14, and 28 days. Participants were provided with access to MK&M and left to use the program as they wished.

### Outcome Measures

Outcome measures were collected using Jisc Online Surveys or via the MK&M platform at baseline, 10 weeks, and 20 weeks. The primary outcome of the SMILE-K trial was the Patient Activation Measure (PAM-13), which assesses an individual’s knowledge, skills, and confidence in managing their health care [31] and ability to effectively self-manage [32], and it has been validated in CKD [33]. The PAM-13 is scored from 0 to 100 and can be categorized into 1 of 4 levels: levels 1 and 2 indicate *low activation*, and levels 3 and 4 indicate *high activation*.

### Sociodemographic

Sociodemographic data, including age, sex, ethnicity, and social deprivation (postcode), were collected. Clinical data, including eGFR, etiology of renal disease, other health conditions, hemoglobin, and albumin, were extracted from medical records.

### Outcomes: Use and Usability Data

Access to and use data of MK&M were collected, alongside perceived usefulness (scored from 0 [not useful at all] to 10 [very useful]) of MK&M content and features. The frequency and length of time spent on MK&M sessions, health and symptom tracker use, and use of other MK&M features (eg, goal setting) were collected via the MK&M platform. Perceived usefulness of MK&M content and features were collected via web-based surveys. Participants were asked to rate how useful they found the MK&M sessions and features out of 10, where 0 was “not useful at all” and 10 was “very useful.”

### Qualitative Interviews

#### Think-Aloud Interviews

Individual think-aloud interviews were conducted to explore PPI participants’ use and engagement with MK&M. Think-aloud interviews involve participants thinking out loud while performing given tasks or recalling their thoughts immediately following the completion of a task [34]. This provided a reflection of participants’ thoughts and reasoning process in real time and was used to assess usability by determining how users interacted with and felt about using the MK&M program [35]. A standardized protocol for think-aloud interviews was followed [34]. As think-aloud interviews generate a wealth of data, it is suggested that a sample of 5 participants will identify 80% of usability problems [35] and yield meaningful results [36]; thus, this was the target sample. Participants were provided with log-in details to MK&M and asked to vocalize out loud their thoughts, actions, and encounters with MK&M. The researcher kept prompts to a minimum. Interviews were conducted via Zoom (Zoom Communications, Inc), with participants sharing their screens, and were audio-visually recorded and transcribed verbatim.

#### Semistructured Interviews

Individual semistructured interviews were used to explore participants’ perceptions and experiences of using the MK&M program during the SMILE-K trial. An interview schedule was developed through familiarization with the current literature and in consultation with PPI partners (different participants from those included in the think-aloud interviews). Topics explored included navigation, functionality, access, engagement, and use of MK&M, alongside other areas of interest. For this qualitative substudy, the target sample was 20 to 30 participants based on information power (an established concept to guide adequate sample size for qualitative studies) [37] and available resources. Purposive sampling was used to capture a diverse range of perspectives and experiences. Participants were invited via email and interviewed via telephone at 10 weeks or 20 weeks following randomization to receive MK&M, and interviews were expected to last approximately 60 minutes. Interviews were audio-recorded and transcribed verbatim by a professional transcription service. All interviews were conducted by one researcher (CJL).

#### Reflexivity

CJL is an experienced qualitative researcher and had no previous relationship with the participants before study commencement. The researcher kept a reflexivity diary, documenting their thoughts and comments during and following participant interviews (both types) and during data analysis. The reflexivity diary aided the researcher to explore and examine their positionality and how it may inform their interpretation of the data generated.

### Data Analysis

#### Statistical Analysis

Unless otherwise stated, data are presented as mean (SD), median (IQR), or n (%). Descriptive and frequency analyses were used to assess uptake and use metrics. Frequency analysis identified the frequency and length of time spent on MK&M sessions. Time is presented as mm:ss, unless otherwise stated. The chi-square analysis compared differences in use metrics at 20-week follow-up between patients with “low” or “high” activation at baseline. Statistical significance was accepted as *P*<.05. Data were analyzed using SPSS 28 (IBM SPSS Statistics).

#### Qualitative Data Analysis

All identifiable information, such as individuals’ names and personal details, was removed from the completed transcripts. NVivo (version 12; QRS International) was used to manage and store data, which were analyzed according to the principles of interpretive reflexive thematic analysis using the approach described by Braun and Clarke [38] to identify and report themes. One researcher (CJL) read the complete dataset to familiarize themself with the content and identified initial codes, followed by review, revision, and determination of final codes. Each script was coded independently by one researcher (CJL), checked by another researcher (REB), and cross-referenced against the codebook developed. Potential themes were identified by one researcher (CJL), which were reviewed and refined with another researcher (REB), and definitions of themes were determined. The researcher developed a codebook to ensure that codes were applied consistently and systematically, and a detailed diary documenting the processes undertaken to provide context and understanding of decisions made during data analysis. Data are presented as direct quotes with participant ID, sex, and age.

## Results

The SMILE-K trial demonstrated that the MK&M program improved patient activation in those who used the resource compared to those who used the standard care, although the overall effect was nonsignificant [10]; the greatest benefits were seen in those with low levels of patient activation at baseline.

### Participant Characteristics

In total, 420 participants were recruited into the SMILE-K trial; of which, 280 (66.7%) participants were randomized into the intervention group and were subsequently provided access to the MK&M program (mean age 59.4, SD 13.6 years; n=161, 57.5% male; n=254, 90.7% White British; eGFR: mean 38.4, SD 17.1 mL/min/1.73 m^2^) and are included in the analyses. Intervention group participant characteristics are displayed in Table 1.

**Table 1:** Self-Management Intervention through Lifestyle Education for Kidney health (SMILE-K) trial intervention group participant characteristics for those assigned to the intervention group and those who activated their My Kidneys & Me (MK&M) account.

Characteristics		Intervention group (n=280)	Activated MK&M account (n=225)
Age (y), mean (SD)		59.4 (13.6)	59.0 (13.6)
Sex (male), n (%)		161 (57.5)	127 (56.4)
**Ethnicity, n (%)**			
	Black	4 (1.4)	4 (1.8)
	Other ethnic group	5 (1.8)	4 (1.8)
	Other White	8 (2.9)	6 (2.7)
	South Asian	8 (2.9)	4 (1.8)
	White British	254 (90.7)	207 (92)
**Education, n (%)**			
	None	4 (1.4)	2 (0.9)
	Primary school	1 (0.4)	0 (0)
	High school	54 (19.3)	46 (20.4)
	College	90 (32.1)	71 (31.6)
	University	87 (31.1)	71 (31.6)
	Other trade or vocational qualification	39 (13.9)	33 (14.7)
**Occupation**, **n (%)**			
	Retired	115 (41.1)	96 (42.6)
	Employed	145 (51.8)	115 (53)
	Unemployed	6 (2.1)	5 (2)
	Other (disabled or long-term sick leave)	4 (1.4)	2 (1)
**Blood pathology, mean (SD)**			
	eGFR^a^ (mL/min/1.73 m^2^)	38.4 (17.1)	37.5 (16.0)
	Creatinine (mg/100 mL)	181.9 (72.5)	184.5 (73.2)
	Hemoglobin (mg/100 mL)	128.0 (18.3)	127.1 (18.4)
	Albumin (mg/100 mL)	40.4 (6.3)	40.5 (6.6)
	ACR^b^ (mg/g)	46.6 (73.6)	54.4 (83.5)
	HbA_1c_^c^ (%)	41.7 (15.7)	41.1 (16.6)
	Urea (mg/100 mL)	12.2 (5.9)	12.6 (6.3)
	CRP^d^ (mg/L)	9.3 (27.8)	7.4 (18.7)
Systolic blood pressure (mm Hg), mean (SD)		138.9 (19.2)	139.2 (19.6)
Diastolic blood pressure (mm Hg), mean (SD)		78.5 (11.3)	78.8 (11.8)
**Comorbidities**			
	Number of comorbidities, mean (SD)	2.5 (1.5)	2.5 (1.6)
	Hypertension, n (%)	209 (74.6)	164 (72.9)
	Type 2 diabetes, n (%)	57 (20.4)	44 (19.6)
	Heart problems, n (%)	55 (19.6)	46 (20.4)
	Blood vessel or circulation problems, n (%)	49 (17.5)	40 (17.8)
	Lung or breathing problems, n (%)	71 (25.3)	53 (23.6)
	Joint, bone, or muscle problems, n (%)	109 (38.9)	88 (39.1)
	Depression, anxiety, or other mental health problems, n (%)	66 (23.6)	55 (24.4)
Taking prescribed medications, n (%)		272 (97.1)	220 (97.8)
BMI (kg/m^2^), mean (SD)		29.4 (7.1)	29.0 (6.8)
PAM-13^e^ score, mean (SD)		61.8 (14.8)	62.1 (14.9)
Low activation, n (%)		100 (35.7)	76 (33.8)

^a^eGFR: estimated glomerular filtration rate.

^b^ACR: albumin to creatinine ratio.

^c^HbA_1c_: hemoglobin A_1c_.

^d^CRP: C-reactive protein.

^e^PAM-13: Patient Activation Measure-13.

### Intervention Access and Use

In total, 225 participants activated their MK&M account across the 20-week study period; of which, 223 (99.1%) activated their account within the first 10 weeks. Over the 20-week study period, only 15 (6.7%) participants accessed their MK&M account once. A total of 63 (28%) participants accessed their MK&M accounts between 2 and 5 times, 40 (17.7%) participants accessed 6 to 10 times, 37 (16.4%) participants accessed 11 to 20 times, and 70 (31.1%) participants accessed >21 times over the 20-week study period. The median number of log-ins per person over 20 weeks was 10.0 (IQR 4.0-28.0); for the first 10 weeks, the median number of log-ins was 5.0 (IQR 1.0-17.0). Over the 20-week study period, the median time per log-in was 12:35 (IQR 6:51-24:55) minutes; for the first 10 weeks, the median time per log-in was 12:07 (IQR 7:16-24:32) minutes.

Individuals with low levels of patient activation (PAM-13) logged into the MK&M program a median of 4.0 (IQR 1.0-11.0) times in 10 weeks and a median of 9.0 (IQR 3.3-19.5) times in 20 weeks. Individuals with high levels of patient activation logged into MK&M a median of 6.0 (IQR 2.0-20.0) times in 10 weeks and a median of 11.0 (IQR 4.5-29.0) times in 20 weeks. Those with low patient activation spent a median time on MK&M per log-in of 14:04 (IQR 7:37-29:50) minutes, while those with high patient activation spent a median of 11:29 (IQR 6:40-23:08) minutes on the program per log-in across 20 weeks. No significant differences were observed between the two groups for total number of log-ins (*P*=.78) and time spent on the program per log-in (*P*=.45).

### Session Access and Use

Table 2 displays learning and action (“How to...”) sessions by total number of times accessed and total duration spent on each session. Overall, “The kidneys” session was accessed by most participants (154/225, 68.4%), followed by the “Kidney disease” (145/225, 64.4%) session. Among the 225 participants, the most viewed learning sessions were “Eating a healthy balanced diet” with 107 (47.6%) participants and a median of 15 (IQR 15-17) views and “Managing my symptoms” with 95 (42.2%) participants and a median of 15 (IQR 15-17) views. The most viewed action session was “How to move more and be active,” with 104 (46.2%) participants and a median of 13 (IQR 13-18) views. The least viewed sessions were “Looking after my well-being” (n=82, 36.4%; median 7, IQR 7-9 views) and “How to look after my well-being” (n=67, 29.8%; median 6, IQR 5-9 views).

**Table 2:** The 20-week My Kidneys & Me (MK&M) usage metrics and perceived usefulness for learning and action (“How to ...”) sessions.

Sessions			Participants, n		Number of times session accessed, median (IQR)		Time spent on session (mm:ss), median (IQR)		Perceived rating of usefulness (out of 10)		
									Values, mean (SD)		Values, median (IQR)
Welcome session			165		2 (2-3)		02:53 (01:14-04:48)		—^a^		—
**Learning sessions**											
	The kidneys	154		7 (7-9)		05:54 (02:52-08:20)		8.6 (2.0)		9 (8-10)	
	Kidney disease	145		12 (11-16.5)		10:57 (06:23-16:37)		8.7 (1.9)		10 (8-10)	
	Kidney disease and general health	133		8 (8-12)		10:52 (05:40-20:33)		8.4 (2.2)		9 (7-10)	
	Treatment options available	116		13 (13-15)		09:00 (05:00-13:52)		8.3 (2.2)		9 (7-10)	
	Reducing my health risks	106		10 (10-13)		07:52 (03:06-14:16)		8.2 (2.2)		9 (7-10)	
	Moving more and being active	103		11 (11-14)		06:16 (02:40-10:28)		8.2 (2.1)		9 (7-10)	
	Keeping my muscles healthy	99		10 (10-13)		08:28 (04:27-13:45)		8.0 (2.3)		9 (7-10)	
	Eating a healthy balanced diet	107		15 (15-17)		08:05 (02:46-15:28)		8.0 (2.3)		9 (7-10)	
	Managing my symptoms	95		15 (15-17)		08:47 (03:34-13:00)		8.1 (2.2)		9 (7-10)	
	Improving my sleep quality	93		7 (7-8)		04:27 (01:45-07:14)		—		—	
	Looking after my well-being	82		7 (7-9)		03:20 (01:37-05:39)		8.1 (2.4)		9 (7-10)	
**Action sessions: “How to...”**											
	Get started	148		4 (4-7)		01:38 (00:55-02:33)		7.8 (2.5)		9 (7-10)	
	Set my own goals	117		12 (11-14.5)		07:50 (03:20-18:16)		7.3 (2.7)		8 (5-10)	
	Move more and be active	104		13 (13-18)		07:21 (03:16-12:51)		7.5 (2.8)		9 (6-10)	
	Keep my muscles healthy through strength training	95		9 (6-12)		05:12 (01:40-10:37)		7.4 (2.8)		8 (6-10)	
	Manage my health	82		6 (6-7)		02:51 (01:32-04:57)		7.4 (2.6)		8 (5-10)	
	Get the most from my health care	77		12 (11-12.5)		05:32 (02:50-09:40)		7.6 (2.5)		8 (6-10)	
	Eat a healthy, balanced diet	77		12 (11-12.5)		06:15 (02:46-09:49)		7.6 (2.7)		9 (7-10)	
	Manage my symptoms	83		6 (6-6)		02:31 (01:32-04:06)		7.4 (2.6)		8 (6-10)	
	Improve my sleep quality	69		5 (5-6)		02:21 (00:52-04:37)		—		—	
	Look after my well-being	67		6 (5-9)		05:34 (02:03-10:51)		7.7 (2.6)		9 (7-10)	

^a^Not available.

Participants spent the longest time on the “Kidney disease” (median 10:57, IQR 06:23-16:37) and the “Kidney disease and general health” (median 10:52, IQR 05:40-16:37) learning sessions; “How to set my own goals” (median 07:50, IQR 03:20-18:16) and “How to move more and be active” (median 07:21, IQR 03:16-12:51) were the action sessions that the participants spent the longest on.

### Goal-Setting Features

In total 40 (18%) participants used the goal-setting and decision-maker (action planner) tools. Of those, 18 (n=40, 45%) participants set more than one goal, with 28% (5/18) setting 5 or more goals. Physical activity (23/40, 57%) and weight loss goals (22/40, 55%) were the most commonly set goals by participants, with more than half of goal-setting users creating action plans for these goals. Goal setting was considered one of the most useful trackers (mean score 7.3, SD 3.0; median score 8.5, IQR 5-10).

### Health Trackers Access and Use

Of the 225 participants, a quarter of participants (n=56, 24.9%) used the blood pressure tracker and 42 (18.7%) used the cholesterol tracker. A total of 26 (11.6%) participants used the smoking tracker, despite only 9 (4%) reporting smoking. The healthy eating and shape trackers were used by 53 (23.6%) and 49 (21.8%) participants, respectively.

### Physical Activity and Exercise Trackers Access and Use

A total of 96 (42.7%) participants tracked their daily step count; of which, 55% (n=53) provided self-reported data and 28% (n=27) imported data from their Fitbit, 11% (n=10) from Google, and 6% (n=6) from a Garmin device. The mean number of days the steps were tracked was 74.3 (SD 50.2; median 69, IQR 22-125), with the mean number of daily steps recorded of 5952.5 (SD 5952.5; median 6189, IQR 2321-8576). Among the 225 participants, 31 (13.8%) used the strength training tracker. The mean duration of sessions was 44.0 (SD 29.2; median 30, IQR 30-52) minutes, with the mean number of repetitions performed per session of 145 (SD 79; median 100, IQR 100-200). The median effort reported per session was 7 (IQR 6-8) out of 10.

### Symptom Tracker Access and Use

The symptom tracker was used by 50 (22.2%) participants, with participants tracking symptoms for a mean of 29.3 (SD 58.7) times (median 11, IQR 3-26 times). Of those, sleep disturbances and tiredness were the symptoms tracked by most number of participants (n=36, 72% and n=35, 70%, respectively), with participants tracking the symptoms for a mean of 3.9 (SD 5.8; median 2, IQR 1-4) and 4.5 (SD 7.5; median 2, IQR 1-4) times, respectively. Bone or joint pain and muscle loss were the most frequently tracked symptoms, with participants tracking for a mean of 4.8 (SD 8.5; median 2, IQR 1-4) and 4.8 (SD 8.8; median 2, IQR 1-3) times, respectively. Table 3 displays symptom tracker use data.

**Table 3:** The 20-week My Kidneys & Me (MK&M) use metrics and perceived usefulness for symptoms, health, and physical activity trackers.

			Participants, n (%)		Number of times the tracker is used					Perceived rating of usefulness (out of 10)			
					Values, mean (SD)		Values, median (IQR)		Values, mean (SD)			Values, median (IQR)	
**Symptoms (n=225)**			50 (22)		29.3 (58.7)		11 (3-26)		7.0 (2.5)			8 (5-9)	
	Itching (n=50)	34 (68)		3.4 (4.7)		2 (1-3)							
	Sleep disturbance or insomnia (n=50)	36 (72)		3.9 (5.8)		2 (1-4)							
	Loss of appetite (n=50)	23 (46)		3.7 (5.0)		2 (1-4)							
	Feeling tired (n=50)	35 (70)		4.5 (7.5)		2 (1-4)							
	Pain in bones or joints (n=50)	33 (66)		4.8 (8.5)		2 (1-4)							
	Poor concentration or mental alertness (n=50)	26 (52)		3.2 (4.8)		2 (1-3)							
	Female participants: loss of libido; male participants: loss of libido or erectile dysfunction (n=50)	22 (44)		3.3 (5.2)		2 (1-2)							
	Loss of muscle strength or power (n=50)	24 (48)		4.8 (8.8)		2 (1-3)							
	Shortness of breath (n=50)	29 (58)		4.5 (7.8)		2 (1-3)							
	Cramp or muscle stiffness (n=50)	30 (60)		4.4 (7.2)		2 (1-3)							
	Restless legs or difficulty keeping the legs still (n=50)	28 (56)		2.9 (4.5)		2 (1-2)							
	Feeling cold (n=50)	29 (58)		3.2 (4.4)		2 (1-4)							
	The need to urinate more often (night or day; n=50)	33 (66)		4.0 (6.2)		2 (1-3)							
**Physical activity (n=225)**										7.5 (2.5)			8 (5-10)
	Number of days tracked	95 (42)		74.3 (50.2)		69 (22-125)							
	Daily steps counted	96 (43)		5952.5 (4149.9)		6189 (2321-8576)							
Strength training (n=225)			31 (14)		8.7 (9.5)		5 (1-15)		7.3 (3.0)			8 (5-10)	
Goal setting (n=225)			40 (18)		2.3 (2.1)		1 (1-3)		7.0 (2.8)			8 (5-10)	
Heathy eating (n=225)			53 (24)		—^a^		—		7.3 (2.7)			8 (5-10)	
Body shape (n=225)			49 (22)		—		—		6.8 (2.6)			8 (4-9)	
Cholesterol (n=225)			42 (19)		—		—		6.8 (2.8)			7 (4-10)	
Blood pressure (n=225)			56 (25)		—		—		7.6 (2.4)			8 (5-10)	
Smoking (n=225)			26 (12)		—		—		6.6 (3.2)			8 (4-10)	

^a^Not available.

### Perceived Usefulness of MK&M Sessions and Features

Tables 2 and 3 display the ratings of perceived usefulness for MK&M sessions and features. All sessions scored a mean ≥7 (median ≥8) out of 10. “Kidney disease” was considered the most useful session, with participants rating it a mean of 8.7 (SD 1.9; median 10, IQR 8-10) out of 10, followed by “The kidneys” (mean 8.6, SD 2.0; median 9, IQR 8-10). “Eating a healthy balanced diet” was perceived to be the least useful learning session, with participants rating it a mean of 8.0 (SD 2.3; median 9, IQR 7-10). The action session perceived to be the most useful was “How to look after my well-being,” rated a mean of 7.7 (SD 2.6; median 8.5, IQR 7-10) out of 10. The session perceived to be the least useful was “How to set my own goals,” rated a mean of 7.3 (SD 2.7; median 8, IQR 5-10) out of 10.

The cholesterol, strength training, and physical activity trackers were perceived to be the most useful trackers, with mean ratings of 7.7 (SD 2.4; median 8, IQR 5-10), 7.5 (SD 2.6; median 8, IQR 5-10), and 7.3 (SD 2.8; median 8, IQR 5-10) out of 10, respectively. The smoking tracker was perceived to be the least useful tracker, with a mean rating of 6.7 (SD 2.9; median 7, IQR 4-10) out of 10.

### Participant Experiences of Using MK&M

In total, 6 PPI partners participated in a think-aloud interview (n=3, 50% male; mean age 68.3, SD 9 y). Of the 36 intervention group participants invited to be interviewed, 31 (86%) responded, and 27 (75%) individuals were interviewed (mean age 61.7, SD 9.2; range 38-76 years; n=16, 59% male; n=25, 93% White British; mean eGFR 34.1, SD 11.7 mL/min/1.73 m^2^). A total of 34 interviews were conducted, with 7 participants interviewed twice at 10 and 20 weeks. Interviews lasted an average of 60 (range 32-96) minutes. Participant characteristics are displayed in Table 4.

**Table 4:** Interview participant demographics.

Participant ID	Age (y), mean	Sex	eGFR^a^ (mL/min/1.73 m^2^)	Ethnicity	Employment status	Activation level (PAM-13^b^)	Interviews, n
SS^c^01	38	Female	45.7	White British	Yes	2 (low)	1
SS02	66	Female	28.4	White British	Retired	2 (low)	1
SS03	71	Female	37.2	White British	Yes	4 (high)	1
SS04	45	Female	57.6	White British	Yes	3 (high)	1
SS05	61	Female	58.8	White British	Retired	4 (high)	1
SS06	55	Female	35.9	White British	Yes	2 (low)	1
SS07	55	Male	20	White British	Yes	2 (low)	1
SS08	54	Male	29.8	White British	Yes	3 (high)	1
SS09	69	Male	24.1	White British	Retired	2 (low)	1
SS10	60	Female	59.2	Other mixed	NR^d^	1 (low)	1
SS11	59	Male	47.5	White British	Yes	4 (high)	1
SS12	72	Male	33	White British	Retired	3 (high)	1
SS13	57	Female	25.6	White British	Retired	4 (high)	2
SS14	57	Male	35.3	Other White	Yes	3 (high)	1
SS15	67	Male	30.1	White British	Retired	4 (high)	2
SS16	68	Male	38.2	White British	Yes	4 (high)	1
SS17	69	Female	26.9	White British	Retired	3 (high)	2
SS18	60	Male	41.4	White British	Retired	4 (high)	1
SS19	71	Male	34.5	White British	Yes	3 (high)	2
SS20	71	Male	35.7	White British	Yes	3 (high)	1
SS21	71	Male	19.1	White British	Retired	1 (low)	1
SS22	55	Female	23.4	White British	Yes	1 (low)	1
SS23	54	Female	21.9	White British	Yes	3 (high)	2
SS24	72	Male	37.5	White British	Retired	2 (low)	1
SS25	76	Male	16.7	White British	Retired	3 (high)	2
SS26	51	Male	32.1	White British	Yes	2 (low)	2
SS27	62	Male	25.5	White British	Yes	3 (high)	1
TA^e^01	55	Female	Stage 3-4	White British	Yes	—^f^	1
TA02	78	Male	Stage 3-4	White British	Retired	—	1
TA03	74	Female	Stage 3-4	White British	Retired	—	1
TA04	71	Male	Stage 3-4	White British	Retired	—	1
TA05	57	Female	Stage 3-4	White British	Yes	—	1
TA06	75	Male	Stage 3-4	White British	Retired	—	1

^a^eGFR: estimated glomerular filtration rate.

^b^PAM-13: Patient Activation Measure-13.

^c^SS: semistructured.

^d^NR: not reported.

^e^TA: think-aloud.

^f^Not available.

### MK&M Use and Engagement

#### Personal and Tailored Information and Support

Having a self-management program specifically designed for people with CKD made participants feel personally involved and empowered to manage their condition, encouraging them to engage with the MK&M program. Being able to tailor and personalize the program to suit their needs was considered an important facilitator:

It does provide a lot of information...it is quite handy to have that as a central point and it’s handy to be able to get sort of information without having to go searching and digging around on the internet.

Participant SS08; male; 54 y; high activation

I’ve learnt a lot more about kidney function and how it relates to me in particular. That’s been the most important thing I think I’ve had on this program, and that’s learning a little bit about how kidneys are supposed to function and what happens when they don’t.

Participant SS15; male; 67 y; high activation

I’m just looking at the last one I was interested in, which is set up device. I presume that’s actually a crucial one, because that’s where you’re going to input most of your data, to set the program running for you personally.

Participant TA04; male; 71 y

Although some participants believed that the “Ask the Expert” feature was a good option to enable a timely response from a specialist kidney health care professional, some felt that it was impersonal and that the person responding to questions would not know their medical history or personal circumstances:

Ask the expert. Yeah, I like that, I like the thought that if I do have a worry, then provided someone does respond within two days.

Participant TA05; female; 57 y

I thought that was a fascinating feature because if you have to wait six months or something to see a doctor, they only have x amount of time, if there’s something you specifically wish to know I think it’s the most wonderful thing.

Participant SS24; male; 72 y; low activation

Obviously the experts won't be able to—won't know anything about my individual case—so whether it would be worth asking them questions I don’t know.

Participant SS16; male; 68 y; high activation

#### Using at Own Pace and Leisure

Participants found that being able to use MK&M at a time that was convenient for them and working through the program at their own pace helped them to engage with it; this helped participants feel like it was something they wanted to do rather than something they had to do. As many participants worked, evenings were considered the best time for them to use MK&M, as this was when they had the most time to spend on the program. Some participants used their lunch breaks at work or commuting time to complete some of the sessions:

In my own time’s good because if it was OK you’ve got to do it by such and such a time it’s like being back at school, no thank you, I’d rather do it at my own pace...I’d rather do it when I’m relaxing because I can take in more information then.

Participant SS10; female; 60 y; low activation

I tend to do it after I’ve finished on the computer, after I finish my work and everything, about, hmm, five time? Just to sort of unwind before everyone comes home.

Participant SS22; female; 55 y; low activation

I did it in big blocks I think really, if I had nothing on in the evening I thought well I’ll just have a look at that program and then once I started reading I just carried on reading.

Participant SS23; female; 54 y; high activation

#### Perceived Time Spent on MK&M

The time participants reported spending on MK&M varied. Participants reported spending large periods on the program in one go and then not engaging with it for a period. Many participants wanted to dedicate time to MK&M as they felt that they were learning more about their health and CKD, and so they would try to find time in their busy schedules to do this, but often this did not translate into practice:

I could spend an hour on it without even realising I’ve been on it an hour...because you’re learning, you’re not just sitting down playing games, you’re actually learning something.

Participant SS10; female; 60 y; low activation

Not that often but when I do I end up doing two or three sessions at a time. So I tend to do it in bulk instead of just one at a time.

Participant SS03; female; 71 y; high activation

I’ve not done it for a few weeks, I sort of let it go, but I did do it at first, maybe not every day, but I certainly—I tried to work through—I did it quite a bit at first but I haven’t really used it for a few weeks.

Participant SS05; female; 61 y; high activation

It’s like a lot of these things, it’s having that time to sit down and look at them all I think, particularly unfortunately me because I was looking at this over the Christmas period, in lots of ways I didn’t quite have the available time to follow that up with the booster sessions.

Participant SS02; female; 66 y; high activation

#### Engaging With MK&M Content

The educational learning and “How to...” action sessions were reported to be the most useful, accessed, and engaged with components on MK&M. Participants perceived these to be interesting and comprehensive:

When I started looking at it, reading through it and everything like that, I was saying oh that’s interesting...they were all very useful, as I say, the goal setting one made me think that would be very useful for people who haven’t came across before.

Participant SS27; male; 62 y; high activation

Very informative. Absolutely loved it...very interesting, very detailed and trying to do things and then you’re going on to the extra booster sessions.

Participant SS04; female; 45 y; high activation

Most participants perceived the trackers to be useful; some reported tracking aspects of their health and physical activity before participating in the trial. Having graphs to visually display their data was considered helpful. Participants felt that the trackers helped to incentivize them to make changes to their health and lifestyle behaviors or to make them more accountable for their current habits. A small number of participants could not relate to the goal-setting features and trackers and became disinterested in them:

The fact that there’s a tracker would be quite useful. I mean I do do all this but I don’t sort of do graphs or anything. So I mean that might be quite helpful.

Participant SS12; male; 72 y; high activation

The tracker’s great because it does incentivise you, definitely, I always want to try and get – I don’t particular – I fill the tracker in every morning and I like to get to what I’ve set myself for.

Participant SS07; male; 55 y; low activation

I’m not a great one for all this kind of target-setting and things that was coming across in some of them. It’s just not something that I’m very good at or very interested in.

Participant SS14; male; 57 y; high activation

Several participants reported not engaging with the chat forum because they did not feel comfortable sharing personal information or communicating with strangers online. Other participants were receptive to the chat forum and found the peer support received beneficial:

I’ve sort of like put a couple of comments and that on there and I’ve got some really good feedback...somebody that knows what I’m going through and understands what I’m going through and they’re giving you more motivation.

Participant SS04; female; 45 y; high activation

The chat forum, I know it’s there, I don’t like chats...I just don’t like communicating online, it’s just not something I’ve done, it’s something I’ve never done.

Participant SS18; male; 60 y; high activation

### MK&M Usability

#### Perceived Accessibility of MK&M

Most participants did not experience any issues accessing MK&M and found it relatively simple to access, even those who described themselves as “technophobes.” Some expressed difficulties initially but managed to access the program after a couple of attempts:

I didn’t find that too difficult really...So we click on any of these blue things and get on with it? Do we double click or...?There you go. ...Technology. Completed?

Participant TA04; male; 71 y

The first couple of times I had trouble logging in because the home page went to, it prefilled in some of the login information which was incorrect...it took us a while to actually get round that as to realise that it was that pre-populated field which was wrong.

Participant SS15; male; 67 y; high activation

A small number of participants reported not receiving their log-in details or not finding them until after a period in their junk mail, resulting in participants not being able to use or engage with MK&M as they might have done if there had not been issues receiving their log-in details:

I only logged on at the end of last week. I didn’t realise that the login had gone to junk mail and I’ve been busy doing other things and I haven’t bothered to check it out, unfortunately. So I’m not as far down the learning curve as I hoped.

Participant SS13; female; 57 y; high activation

#### Perceived Interactivity and Presentation of Information

Participants discussed their views on the presentation and interactivity of MK&M. Many reported liking the presentation of the information, particularly when it was presented as an infographic, but a few people would have liked more font formatting to highlight key information and improve readability. The color palette had mixed opinions; some felt that it was attractive and eye-catching, whereas others felt that it could have been more colorful. Several participants proposed suggestions to improve the interactivity, such as presenting the information in a variety of ways to support different learning styles, to encourage people to better engage and interact with MK&M:

I like the way it’s set out, it’s very easy. Everything is within one window and you click on one and it pops up with more information. I like that. I think it’s a very well set out platform.

Participant SS13; female; 57 y; high activation

Some people do switch off quite early on. But all the graphics and things, and making things interesting and bold. Keep them going...The graphics are fine in blue and white. Stand out beautifully, but when it comes to the writing, the introduction, etcetera, they need to be bolder for me.

Participant TA03; female; 74 y

You could make it nice and bright and colourful and what have you, but to be honest does it really make any difference whether something’s in red or yellow or what have you, in the grand scheme of things it’s – you know – it’s easy to read.

Participant SS05; female; 61 y; high activation

I think if people are interacting, like you’ve got a lot of information, but if you make people think before they read it, it would just be more interesting...because everyone thinks about things in different ways, and it’s more likely to go in as well if you interact with thing.

Participant TA06; male; 75 y

#### Logical Navigation and Progression

It was perceived that MK&M had a logical flow, and navigation around the program was considered straightforward and coherent. Educational sessions were believed to have a natural order and transition from one topic to the next, making it easy to use and engage with. Although some participants reported following the sessions in the order in which they were set out, others discussed how they chose to start with sessions that they perceived to be of greatest interest to them:

I think as a complete package to go from one bit to the next, yeah, it progresses you from one section onto the next one quite nicely.

Participant SS05; female; 61 y; high activation

It’s quite easy to navigate where you want to get to. You’ve got your headers, so that sort of shows you a bit about where you are. You’ve also got the progress section down the side as well, which tells you how many bits that you’ve done, and how many bits are left.

Participant SS06; female; 55 y; low activation

I tended to come back and I chose a more interesting one initially and then worked through that and then gradually the ones that perhaps you are less interested in I also did, but much later in sequence.

Participant SS25; male; 76 y; high activation

#### Ability to “Dip in and Out”

Participants discussed how the bite-sized information helped break up the sessions into manageable chunks. Having different sections within the program and being able to access MK&M periodically enabled individuals to engage with the program as they chose. Although most participants favored frequent and brief interactions with MK&M, some participants preferred to spend significant periods on the program but more infrequently:

You can just, whenever you’ve got five or ten minutes go on it wherever you are, so I did find that useful.

Participant SS23; female; 54 y; high activation

I tended to look at the booster session, I’d look at it on the tram on the way into work...it was useful, because they were short, I could fit them into little bits of my day. I found that quite helpful. I didn’t have to do it all at once.

Participant SS01; female; 38 y; low activation

I wouldn’t just pop in and pop out because there’s no point. You want to sit down and get something out of the session, so sit down like you’d sit down and read a book for an hour or so.

Participant SS13; female; 57 y; high activation

### Ongoing Engagement With MK&M

#### Desire for Continued Learning

The need to learn more and improve their knowledge of how best to self-manage their CKD was a motivating factor for participants in continuing to engage with MK&M. Participants were hopeful that new information would continue to be released, with alerts and reminders to let them know when this was available:

There’s no way you know everything! There’s always learning to be had and the more knowledge you have with regard to something like this then you know that you’re doing the best that you can to support you move in a positive direction.

Participant SS02; female; 66 y; low activation

I’ve done a couple of big sessions and got through quite a lot of the initial introductory stage. Yeah, it’s quite interesting. Hopefully as I carry on, I’ll find out a bit more and we’ll know what’s good for me.

Participant SS06; female; 55 y; low activation

I haven’t discovered half of what it’s doing yet, but I’m plodding my way through. Rather than sitting in here now I’m, sort of, working my way through just the one at the moment.

Participant SS13; female; 57 y; high activation

#### Dedicating Time to MK&M

Participants felt that they did not have enough time to complete the program within the study period but were keen to keep using MK&M, with many reporting that they planned to look at the sessions that they had not completed in time. Committing time to MK&M was perceived to be important and not overly difficult. Being able to see their progress helped participants to keep track of their progress and pick up where they left off:

The contents is fine, using it is fine, it’s just me finding the time to go through it...it’s been very, very informative, but unfortunately I’ve not been able to keep up with it at the moment.

Participant SS10; female; 60 y; low activation

I don’t think you have to commit loads of time, but you know, being able to commit a bit of time would be good. You can see how far you’ve got, for a start. And it’s nice to know you can carry on from where you’ve left off.

Participant SS06; female; 55 y; low activation

#### Recognizing the Need to Prioritize MK&M

MK&M was not considered a top priority by participants. Although participants expressed being receptive to MK&M and intending to use and complete the program, they felt that competing interests and other commitments were of greater importance:

They don’t consume a lot of time but it’s just actually prioritising things you do isn’t it...I’m not very good at sitting down.

Participant SS02; female; 66 y; low activation

People, like myself, you start off with good intentions and then perhaps you don’t finish it off or then lose interest, perhaps you don’t put it on your top priority. People might think it’s too hard to do anything or some people just don’t want to know.

Participant SS05; female; 61 y; high activation

#### Perceived Relevance as Disease Progresses

It was perceived that MK&M could become more useful in time, particularly as the disease progresses. Many participants felt that some of the features, such as the symptom tracker, were not relevant at the moment, as they did not experience any or some of the symptoms, but they could see how the features would be useful in time. Identifying potential consequences of what could happen as the disease progresses was believed to be empowering:

I think you’ll see with time that this function will become more important.

Participant TA04; male; 71 y

I think helping managing my condition, and it does do that. I think it would be potentially – this sounds awful – if I was sicker, kind of like that would help a lot...from the point of view of identifying “Look, this can be the consequence of having this”...it would be potentially quite empowering.

Participant SSO1; female; 38 y; low activation

## Discussion

### Principal Findings

This mixed methods study explored the use, usability, and user experience of the MK&M program to identify the content and features accessed, used, and valued. The study findings provide a detailed account of how users engage with and experience MK&M and suggest that the program is well-received and used by participants. Interviewing participants about their experiences provides detailed context and explanation of the quantitative data, helping to further understand the use metrics data. The integration of qualitative and quantitative findings allows for a comprehensive evaluation of the use and usability of MK&M, highlighting areas requiring attention and considerations for revising and adapting the program to improve user experience.

The educational sessions were the most valued and engaged content, while the health and symptom trackers were the least used features. The MK&M sessions mostly accessed and used were about kidneys, kidney disease, and how CKD relates to general health. This is not surprising given the need and appetite for kidney education [39]. There is a concerning lack of knowledge about kidneys, with many people not aware of what their kidneys are for or even where they are [40]. For those affected by CKD and its associated effects, being able to understand what to do, make decisions, and take action is imperative for individuals to better self-manage [40]. Interview participants in this study felt that as MK&M was specifically for people with CKD, it made them feel personally involved and empowered to manage their condition. For people with long-term conditions, such as CKD, specifically designed DHIs can empower and motivate them by providing feelings of reassurance and the ability to self-manage their condition [41]. Findings from the main trial evaluating MK&M demonstrated that it was effective at improving people’s knowledge, skills, and confidence in managing their CKD, alongside improving self-management behaviors, particularly for those with low levels of preexisting patient activation [10]. Despite logging in slightly less than those with high patient activation, those with low levels of activation spent longer on MK&M per log-in, with the total time spent on MK&M across the study period comparable between the two groups. This is encouraging as, overall, there is little difference in the engagement with MK&M for low and high-activated patients. Individuals who perceive DHIs as beneficial for their health are more willing to adopt and engage with them [42]. Greater adoption and engagement levels are observed when DHIs are based on individuals’ needs and preferences and are perceived as valuable additions and tools that can enhance individuals’ overall health and well-being [43].

MK&M was well-received by participants, with many reporting that the user interface was easy to use, with clear and logical navigation and appropriate presentation of information. Design features, ease of use, perceived usefulness, and personalization are key factors influencing user experiences [44] and empowering individuals to engage with DHIs [45]. Being able to access and use MK&M at one’s own pace and leisure was identified as an important facilitator for engagement. Having control and autonomy over the type, time, and location of intervention access is considered advantageous over traditional face-to-face care [46]. Moreover, user experience, including usability, acceptability, and satisfaction, has been shown to play a crucial role in the successful implementation and long-term engagement with DHIs [47]. Maintaining interest in the intervention over time has been identified as one of the main challenges of DHIs [48]. Website log-ins, a commonly used indicator of engagement and intervention exposure, often decrease over time [49,50]. We did not observe a decrease in MK&M use over the study, with a similar number of log-ins and time spent on MK&M observed between 0 weeks and 10 weeks, and 10 weeks and 20 weeks. However, as the study period was only 20 weeks, no user metric data were obtained beyond this. Further work is needed to assess the long-term use of MK&M. In addition, evaluating the impact and attrition of MK&M after implementation in real-life settings is required, as evidence suggests that use and engagement with DHIs are greater in trial users compared to “real-life” users [51,52]. Work is planned to explore users’ perspectives of implementation requirements and sustainability of MK&M, alongside embedding MK&M into routine clinical practice.

User engagement with MK&M was much greater than other self-management DHIs, including those for diabetes [53-55], with MK&M participants spending approximately 2.5 hours on the program across the 20-week study period, with some accessing MK&M more frequently and for longer periods. Interview participants reported spending significant periods using MK&M; however, they also expressed a need to dedicate more time and priority to the program, as they felt they had more to gain from MK&M. The learning sessions were more widely accessed and used than the action (“How to...”) sessions, and while it is not explicitly clear why this was the case, participants did highlight not having enough time to engage with all the MK&M content in the study period. Other DHIs have found that there is greater exposure to content which appears earlier in the program [55]; consequently, most users are not exposed to the broader theory- and evidence-based content that goes beyond information giving [55] and is found to be most effective and important for behavior change [56]. Lack of exposure to enough of the intervention content because of poor user engagement can make intervention effectiveness difficult, which is a common issue relating to DHIs [57]. Consideration must be given when implementing DHIs, including MK&M, to ensure that there is adequate and equitable exposure to the different constructs of self-management (ie, medical, emotional, and role). Identification of methods to increase exposure and encourage engagement with the active components of DHIs is required [55]; this may involve health care professionals signposting users to the relevant information and support. Although MK&M provides users with the flexibility to access and use the content in a way that is most useful, use metrics suggest that individuals may work through MK&M in a more structured fashion and perhaps are not exposed to the behavior change theory–driven content. Further work is required to understand approaches to increase exposure to the important self-management and behavior change content that can improve patient outcomes.

Generally, MK&M users had positive experiences of using the program; however, there was ambivalence regarding content and features, which could be explained by personal preference rather than usability issues. Substantial ambivalence has been reported for DHIs, with a diversity of experiences identified that are considered unique to each individual [41]. One notable concept is that some users want to be part of a sharing community while others prefer privacy [41]; the MK&M chat forum had mixed opinions, but despite many perceiving it as not relevant for them, they could see how it might be valuable for someone else. In addition, there were variations in the use and perceived usefulness of the health, symptoms, and physical activity trackers and goal-setting features. Lower engagement with tracking tools has been observed for other self-led programs [58] and where users engage with other digital tracking tools outside of the program [59]. Greater use of DHIs and their trackers to support self-monitoring and management is associated with better outcomes [60-62]; thus, further work on understanding factors influencing the use of the trackers and goal-setting features in MK&M is warranted.

Participants had a desire for continued learning and perceived the relevance of MK&M to be greater with time and disease progression. The use of DHIs is dynamic, and there may be more active periods when users engage more intensively and periods of moderate use or nonuse [63]; however, these few active periods are considered a part of the engagement process and may not mean that participants are not engaged anymore [63]. It is argued that adherence goes beyond using the DHI and relates more to adhering to the intended use that corresponds to desired goals [64]. The ability to create a routine with a DHI and incorporate it into part of your daily life is an important component of behavioral engagement [63]. This is something that participants in our study recognized and were hoping to prioritize and dedicate more to using MK&M. It appears that engagement with MK&M may fluctuate over time and respond to changes in health and disease progression. Raising patient awareness of CKD and its associated complications is essential for optimal management [6]; thus, providing intervention at an early disease stage can enable individuals to become more familiar with and make better sense of the information. Providing insights into users’ health, feelings of empowerment over being in control of their health, and improving skills have been found to facilitate DHI engagement [65]. Understanding the most appropriate time to offer MK&M to people with CKD is needed, and it is currently being explored.

Participants in this study did not experience any difficulties in using MK&M, despite some issues with activating their MK&M accounts. It is important to note that this trial was remote and virtual in nature, and thus, to be able to participate, individuals needed a certain level of digital literacy. Although not the focus of this study, it is necessary to acknowledge that digital poverty exists, with disparities in access to and proficiency in using digital devices and technology. Individuals with poor digital literacy and without access to digital technologies, particularly those with lower socioeconomic status and living in rural areas, may face greater challenges in adopting and using DHIs [66]. Bridging this gap requires conscious efforts to improve digital literacy, provide support and training, and ensure equitable access to technology and internet connectivity [67]. Improving the equity of access for those who are potentially disadvantaged and stand to benefit from MK&M is crucial. Future work should explore how we can improve digital inclusion for disadvantaged and underserved groups.

Primary findings from the SMILE-K trial suggest that when used, MK&M leads to important improvements in patient activation and related CKD self-management behaviors, with greater improvements observed in those with low patient activation at baseline [10]. Findings suggest that there is a need and appetite for DHIs among those with CKD to better manage their health, and MK&M is an appropriate means to deliver kidney health education to improve patient activation and self-management. For this study, MK&M was not prescriptive and provided minimal health care professional contact; participants were provided access to MK&M and left to use the program as they wished. Although there are many factors that affect an individual’s ability to engage with DHIs [68], this work provides a comprehensive evaluation of how users engage with and experience MK&M. In addition, this work highlights key areas that require attention and consideration. The findings of this study will be used to revise and adapt MK&M to improve accessibility, support uptake and engagement, and improve user experience and interaction with the platform.

### Strengths and Limitations

This study has several strengths and limitations. A key strength of this study was the integration of qualitative and quantitative results, which helped to provide a more comprehensive understanding of the use, usability, and user experiences of the MK&M program. The inclusion of qualitative methodology, including think-aloud, enabled us to explore user experiences of the program and provide context to the quantitative findings, giving us a richer depth of insights into how MK&M was perceived and used.

A limitation of the qualitative substudy was the potential bias of the participants recruited, which may limit the generalizability of the findings. Although the participants interviewed generally had positive feedback, it may be that those who had different opinions or negative experiences were not willing to participate. Although the intended sample size was selected using the maximum variation technique, there was self-selection bias as several participants who were invited to be interviewed did not take part. In addition, due to the virtual nature of the trial and the intervention, individuals with low levels of digital literacy were likely disadvantaged, and individuals without access to digital technology were ineligible. Thus, those involved in the main trial and the qualitative substudy had to have had some level of digital literacy to be able to enroll in the study, and thus it is not known how individuals experiencing digital poverty may have used or engaged with MK&M. Further work is needed to improve the equity of DHIs for people from potentially disadvantaged groups, and specific revisions to improve accessibility and engagement for these populations will be undertaken.

The design of MK&M was guided by the user-centered design principles, and the usability testing of MK&M with people living with CKD has ensured that MK&M is accessible and easy to navigate for our target population. However, the study population is fairly homogenous and not necessarily representative of the CKD population. Further work is required to explore how to improve uptake, adoption, and engagement with individuals from disadvantaged and underserved groups.

### Conclusions

Overall, MK&M DHI was well-received and used by participants. The educational content was more widely and frequently used by participants and was perceived to be the most useful component of MK&M. The information provided was considered comprehensive, and its presentation made it easy for users to access and engage with. Our findings demonstrate that people with CKD are capable and willing to use DHIs to improve their kidney health and self-management knowledge and behaviors. Raising awareness of CKD by providing information about CKD and its management via resources such as MK&M is paramount to optimizing care and treatment and will positively impact an individual’s ability to self-manage their health. As better self-management is associated with better health outcomes and lower health care costs, MK&M has the potential to improve CKD health care management. Future trials are needed to evaluate the impact of CKD self-management DHIs, such as MK&M, on delaying disease progression, preventing progression to end-stage kidney disease, reducing cardiovascular morbidity and mortality, preventing unnecessary hospital admissions, and reducing health care costs. Findings will be used to revise the program to improve accessibility, engagement, and user experience. Further work is also required to revise and adapt MK&M to increase uptake and engagement among disadvantaged and underserved groups, who are likely to have the most to gain from using the program. In addition, the identification of real-life use and usability issues will help refine MK&M to improve the content and delivery before clinical implementation. Understanding the implementation strategies and processes required to ensure the successful delivery, reach, adoption, and sustainability of MK&M is warranted, and future implementation trials will be conducted to evaluate the translation of this research into action.

## Abbreviations

CKD: chronic kidney disease
DHI: digital health intervention
eGFR: estimated glomerular filtration rate
MK&M: My Kidneys & Me
PPI: patient and public
RCT: randomized controlled trial
SMILE-K: Self-Management Intervention through Lifestyle Education for Kidney Health

## Data Availability

The data that support the findings of this study are available from the corresponding author upon reasonable request.
